# Effect of open vs. closed kinetic chain exercises in ACL rehabilitation on knee joint pain, laxity, extensor muscles strength, and function: a systematic review with meta-analysis

**DOI:** 10.3389/fspor.2024.1416690

**Published:** 2024-06-03

**Authors:** George M. Pamboris, Kyriakos Pavlou, Eleftherios Paraskevopoulos, Amir A. Mohagheghi

**Affiliations:** ^1^ Department of Health Sciences, School of Sciences, European University Cyprus, Nicosia, Cyprus; ^2^ Department of Physiotherapy, Aegean College, Athens, Greece; ^3^ Laboratory of Biomechanics, Department of Physiotherapy, University of Peloponnese, Sparta, Greece; ^4^ Division of Sport, Health, and Exercise Sciences, Brunel University London, Uxbridge, United Kingdom

**Keywords:** knee, anterior cruciate ligament, open kinetic chain, close kinetic chain, rehabilitation, systematic review

## Abstract

Anterior cruciate ligament (ACL) injuries are common among physically active individuals, often requiring ACL reconstruction (ACLR) for recovery. Rehabilitating these injuries involves determining the appropriate timing for initiating open kinetic chain (OKC) exercises. Although OKC exercises are effective post-ACLR, their use in rehabilitation remains a subject of debate. Therefore, this study aims to conduct a systematic review to determine whether OKC or closed kinetic chain (CKC) exercises result in differences in laxity, strength of the knee extensor muscle group, function, and functional performance in ACL rehabilitation. Five electronic databases were searched for randomized controlled between-group trials (RCTs). Two reviewers independently evaluated the risk of bias using the PEDro scale. We performed a meta-analysis using a random-effects model or calculated mean differences (fixed-effect) where appropriate. Certainty of evidence was judged using the GRADE approach. The systematic literature search yielded 480 articles, of which 9 met the inclusion criteria. The evidence for all outcomes ranged from very low to low certainty. Across all comparisons, inconsistent results were found in outcome measures related to knee function between OKC and CKC exercises post-ACLR. A significant increase in quadriceps isokinetic strength was found in post-ACLR and ACL-deficient knees in favor of OKC exercises at 3 (*p* = 0.03) and 4 (*p* = 0.008) months, respectively. A significant decrease in knee laxity was observed in ACL-deficient knees in favor of OKC at 10 weeks (*p* = 0.01), although inconsistency was noted at 4 months. Finally, a significant decrease in pain was found in favor of early OKC compared to late OKC (*p* < 0.003). Additionally, in ACL-deficient knees, low load resistance training (LLRT) OKC showed no significant laxity difference compared to controls (*p* > 0.05). In contrast, high load resistance training (HLRT) OKC had less laxity than controls at 6 weeks (*p* = 0.02) but not at 12 weeks (*p* > 0.05). OKC exercises appear to be superior to CKC for improving quadriceps strength 3–4 months post-injury, whether as a part of conservative or post-surgery rehabilitation. On the other hand, OKC exercises seem to be either superior or equally effective to CKC for improving knee laxity, thus presenting their importance in being included in a rehabilitation protocol from the initial phase.

**Systematic Review Registration:**

PROSPERO [CRD42023475230].

## 1 Introduction

Anterior cruciate ligament (ACL) injuries are among the most common and debilitating knee injuries that occur in athletes (1). After ACL injury, most patients opt for ACL reconstruction, where aggressive postoperative rehabilitation protocols are often performed (2) to restore knee stability and function, enabling individuals to return to daily activities, including sports, and reducing their risk of developing osteoarthritis (OA) (3). However, surgery cannot succeed without adequate postoperative rehabilitation to optimize outcomes and ensure long-term success (4). There is a high risk of long-lasting functional deficits in muscles crossing the knee joints (5), with postoperative pathological laxity and graft reinjury remaining a concern (6).

One area of interest in ACL rehabilitation is the selection of appropriate exercises that facilitate the recovery of knee stability, strength, and overall function. Two exercise paradigms that have gained attention in recent years are open kinetic chain (OKC) and closed kinetic chain (CKC) exercises (7, 8). OKC exercises allow free movements of the distal joint segment in space without weight-bearing (WB), such as seated leg extensions, terminal knee extension exercises, hamstring curls, and calf pumps (9). A characteristic of OKC exercises includes more isolated muscle activity, thus allowing for more specific muscle strengthening (10, 11). These exercises improve strength and range of motion (ROM), encouraging normal movement patterns (9). OKC exercises, specifically ones promoting knee extension, are believed to be damaging because they can place high strain on the ACL graft or healing ACL, loosening it and increasing knee laxity (12).

While OKC exercises allow movements of the distal joint segment without WB, CKC exercise is when the distal segment is fixed, prohibiting free movement of that segment, such as squats and lunges (10). Movement still occurs in each joint of the system participating in the chain. Unlike an OKC exercise, CKC exercise promotes the co-contraction of muscles to stabilize and control joint movements (13). Multiple muscle groups are typically activated around the joint instead of contracting only one group of muscles (13).

There are conflicting findings regarding the effect of open OKC exercises on knee laxity after ACL reconstruction (ACLR). Nelson et al. (12) and Perriman et al. (14) reported no significant difference in knee laxity between OKC and CKC exercises. This suggests that both exercise types may be equally effective in improving knee stability. Nevertheless, clinical trials *in vivo* have shown that CKC exercises can reduce knee laxity by activating the co-contraction of the quadriceps and hamstring muscles (15) in patients after ACLR.

The general practice regarding the timing of postoperative rehabilitation varies depending on the timing of surgery, choice of graft (autograft, allograft, one- or two-bundle technique), and fixation method (11). Traditionally, CKC exercises have been preferred over OKC exercises for ACLR rehabilitation. This preference was based on the belief that OKC exercises may put more strain on the reconstructed ACL, leading to increased knee pain and laxity compared to CKC exercises (16). Wilk et al. (17) reported that during isotonic OKC knee extension exercises, there is minimal to no hamstring muscle activity, particularly near terminal knee extension (at approximately 40° to 0° of knee flexion), where the amount of quadriceps force produced to extend the knee joint is 3–4 times greater, thus resulting in higher ACL strain. This co-contraction of the quadriceps and hamstrings is important in reducing anterior tibiofemoral shear forces and ACL strain (18). Nevertheless, in many cases, postoperative rehabilitation involving OKC begins relatively soon after surgery, often within the first few days or weeks, since studies have reported that the early incorporation of OKC exercises in the early stages of ACLR rehabilitation does not adversely affect anterior tibial translation compared with the later initiation of these exercises (19–22). Introducing OKC exercises, with a particular emphasis on quadriceps strengthening, could also offer benefits regarding muscle activation, as they have been shown to aid in the recovery of isolated muscle activation (21).

This paper primarily aimed to review and analyze existing literature on the effect of OKC or CKC exercises on laxity, strength of the knee extensor muscle group, function, and functional performance after ACL reconstruction. The secondary aim was to determine whether there were any differences between OKC and CKC exercise protocols after ACL injury/deficiency for these clinical outcomes. Thirdly, we aimed to evaluate the clinical outcomes after ACL reconstruction with early (less than 6 weeks) vs. late (more than 6 weeks) start of OKC exercises of the quadriceps muscles in patients after ACLR. Lastly, we searched for evidence regarding the effectiveness of OKC resistance exercises in function, laxity, and functional performance in ACL-injured individuals.

## 2 Methods

### 2.1 Protocol and guidelines

This systematic review was preregistered in the International Prospective Register of Systematic Reviews (PROSPERO) (Registration number: CRD42023475230). Additionally, it was conducted in accordance with the preferred reporting criteria set out in the Preferred Reporting Items for Systematic Reviews and Meta-Analyses (PRISMA) statement (23).

### 2.2 Eligibility criteria based on the PICOS framework

The primary eligibility criteria were formulated based on the Population, Intervention, Comparison, Outcome, and Study (PICOS) framework (24) and were predefined as follows:

• **Population:** Studies were eligible for inclusion if they recruited adult patients aged ≥15 and ≤60 years with ACL injury and no other pathology in the injured or contralateral limb. Studies that evaluated the effects of OKC/CKC exercises in patients with other comorbidities such as OA, ACL reinjury, total meniscectomy, systemic diseases such as diabetes, rheumatoid arthritis, or other pathologies (cardiorespiratory, neurological, autoimmune diseases) were excluded.
• **Intervention:** Studies that investigated the effects of open and CKC exercises were considered eligible for this review.
• **Comparison:** Studies that compared OKC and CKC or OCK/CKC vs. a non-treatment or standard treatment group were considered eligible for this review.
• **Outcomes:** Studies were considered eligible if they analyzed at least one of the following outcome measures at baseline and final follow-up assessment: (1) quadriceps muscle strength (using dynamometry); (2) function (assessed using self-reported questionnaires); (3) pain with a subjective measurement; (4) functional performance (measured by horizontal, vertical, and triple cross-over jump tests), and (5) anterior knee laxity (measurement using arthrometry, clinical testing or instrumented examination).
• **Study design:** Non-randomized and randomized controlled trials (RCTs) were considered eligible for this review.

The inclusion of predatory journals in literature reviews may have a negative impact on the data, findings, and conclusions. We adhered to the established guidelines for identifying and excluding predatory journals from the findings (25). Articles sourced from open-access journals were assessed to determine if the host was a “predatory journal” by checking if the journal was listed in the Directory of Open Access Journals (DOAJ) and/or was a member of the Committee On Publication Ethics (COPE). If the answer remained unclear, the journal website was reviewed for characteristics of predatory journals (25). Only one paper met these criteria, containing logical inconsistencies and not listed on either DOAJ or COPE. For completeness, we also searched an informal list of predatory journals (https://predatoryjournals.org), which confirmed that the journal was listed there. Thus, the study was excluded.

### 2.3 Search methods

**Figure 1:**
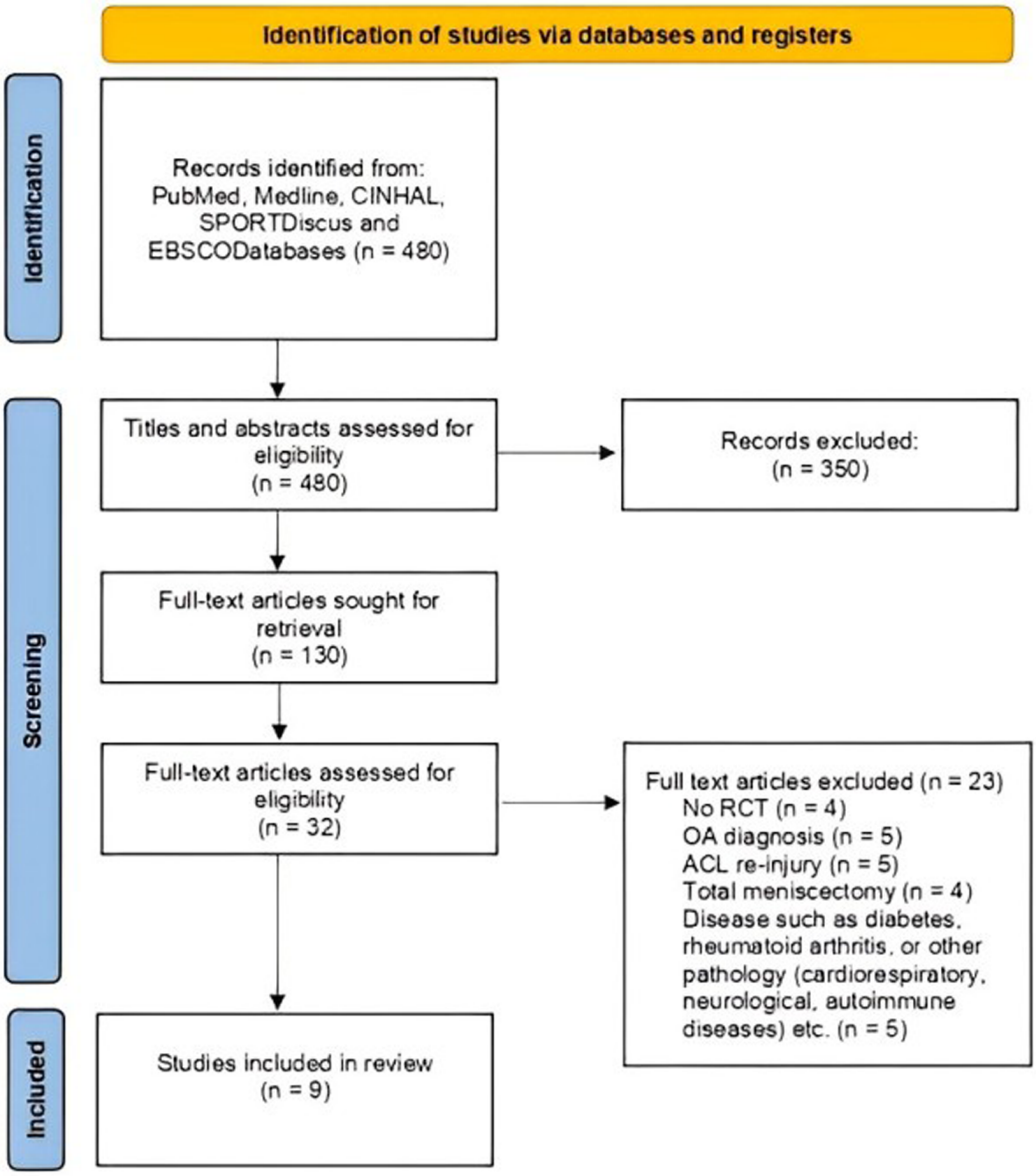
PRISMA flow diagram with search protocol summary.

International electronic databases (PubMed, Medline, CINAHL, SPORTDiscus, and EBSCO) were used for the literature search from March 2005 to March 2024. Figure 1 provides a schematic of the research methodology. These databases were searched using the combinations of the following keywords: (1) “anterior cruciate ligament reconstruction,” “exercise training,” “open chain exercises,” and “closed chain exercises” and (2) “laxity,” “strength,” “pain,” and “function.” The terms were connected with “OR” within each of the two combination groups, and these two search categories were combined using “AND”. Additional searches were subsequently conducted in Google Scholar if full-text articles were not fully available; these allowed for articles to be found on ResearchGate if they were unavailable through the aforementioned electronic databases. Finally, using the full-text articles, reference lists were checked for additional suitable research studies that had not been identified using the previous methods. In case of missing data, authors were contacted via email.

### 2.4 Study selection

Articles were initially screened for eligibility by title and abstract. Two independent reviewers (GP and EP) performed the search and the entire inclusion process using the PICOS framework. Full texts of potentially relevant articles were retrieved for final evaluation. The selection process for the selected studies was conducted by consensus, and when a consensus was not achieved, a third reviewer (KP) was available to assist the process for a final judgment.

### 2.5 Data extraction

One reviewer (GP) collected relevant details independently using a standardized form that collected information regarding participant characteristics, study design, follow-up, interventions (i.e., type, duration, and the number of sessions), comparison group characteristics, and outcome measures; pre- and post-intervention means and standard deviation for any anterior tibial laxity, lower limb strength, pain, function, or functional performance measures; and main within- and between-group results (anterior tibial laxity, lower limb strength, pain, function or functional performance). A second investigator (KP) reviewed all data for accuracy.

### 2.6 Quality assessment and risk of bias

The methodological quality and risk of bias of the included studies were assessed by two reviewers (GP and KP) using the Physiotherapy Evidence Database (PEDro) scale, which determines any potential risks for bias within a study and has been established as a reliable tool for assessing RCTs (26). The scores were confirmed by cross-checking with the scores provided on https://pedro.org.au/. The PEDro scale consists of 10 questions designed to identify potential weaknesses within each study (26). Questions 1 and 2 target participant group allocation and randomization, while questions 5, 6, and 7 explore the types of blinding performed within RCTs (26). The remaining questions (4, 8, 9, and 20) assessed participant characteristics and methods of reporting results (26) and were a crucial focal point when comparing the eligible studies. These items could assist the readers in identifying studies that are likely to be internally valid (items 2–9) and studies with sufficient statistical information to make their results interpretable (items 10–11) (27). The final score of the PEDro may range from 0 (low quality) to 10 (high quality). Studies can be rated as high (≥7/10), moderate (4–6/10), or low quality (≤3/10). A PEDro quality score of <7 indicates a study as having a “high” risk of bias (28).

### 2.7 Data synthesis and analysis

The ReviewManager Version 5.3 software (The Nordic Cochrane Centre, Copenhagen, Denmark) was used to summarize the effects of OKC and CKC on (1) muscle strength, (2) function, (3) pain with a subjective measurement, (4) functional performance, and (5) anterior knee laxity. Subgroup analysis was performed for each outcome measure based on group assignment/intervention. We subjectively categorized studies based on the time of introduction of the OKC/CKC exercises after ACLR into early (<6 weeks) or late (>6 weeks) start times. Follow-up times were either short-term (<12 weeks), medium-term (3–6 months), long-term (6–12 months), or very long-term (>12 months).

Following the recommendations of the Cochrane Handbook for Systematic Reviews of Interventions (29), our quantitative synthesis was conducted using the post means and standard deviations from each selected study for the between-group comparisons. These were either extracted directly from the articles or calculated, if needed, based on the procedures outlined in a previous study (30).

Using the available outcome measures, we calculated standardized means. When data from multiple studies were available, we conducted a meta-analysis to compare standardized mean differences (SMDs) and their associated 95% CI. Our analysis considered variations in clinical settings and assessment methods for joint laxity, muscle strength, pain levels, function, and functional performance. Mean differences (MDs) were calculated and presented to determine the availability of individual study data. At the same time, pairwise meta-analyses with forest plots were performed when two or more studies were accessible, meeting homogeneity criteria (31). Pooled analyses were conducted for studies evaluating the same group, using similar assessment methods for the outcome measures where the recruited participants displayed comparable demographic characteristics, and after leave-one-out sensitivity analyses. An effect size (MD, fixed-effect model) was calculated for outcomes with only one available study. Summary tables presented the results for each outcome. ReviewManager Version 5.3 was used for effect estimates, employing a random-effects meta-synthesis when methodological and setting heterogeneity was assumed between studies. Subgroup analyses were performed for graft types (patellar and hamstrings).

In cases of significant between-group statistical heterogeneity (i.e., *I*^2^ > 75%), meta-analyses were not omitted (31); instead, we also evaluated heterogeneity using sensitivity analyses by excluding studies with unexpectedly large treatment effects and employing a “leave-one-out” exclusion approach. Due to the limited number of studies, assessment of reporting bias using a funnel plot was not feasible. According to Cohen’s criteria, SMD values were classified as small (≤0.20), moderate (between 0.21 and 0.79), and large (≥0.80) (32).

### 2.8 Certainty of evidence

Certainty of evidence was evaluated using the Grading of Recommendations, Assessment, Development and Evaluation (GRADE) (33, 34), and tables were created and exported using the GRADEpro software (https://gdt.gradepro.org/). Based on the criteria below (Table 1), the quality of evidence was classified as very low, low, moderate, or high depending on the presence of risk of bias, inconsistency, indirectness, imprecision, and publication bias (where applicable). Any disagreements were resolved by the involvement of a third investigator (AM).

**Table 1:** Criteria used for grading the certainty of evidence.

GRADE domain	Criteria for downgrading the certainty of evidence using the GRADE methodology
Risk of bias	Certainty of evidence was downgraded by one level if the “low-risk” studies contributed <50% of participants in the pairwise comparison (PEDro score of <7 determined a study as having a “high” risk of bias
Inconsistency	Certainty of evidence was downgraded by one level if (1) the overlap of 95% CIs presented in forest plots was poor; (2) the magnitude and direction of the effect were inconsistent between studies, and (3) the strength of the evidence suggesting substantial heterogeneity (*p*-value from *χ*^2^ test or *I*^2 ^> 50%)
Indirectness	Certainty of evidence was downgraded one level if heterogeneity in population characteristics or interventions was evident
Imprecision	Certainty of evidence was downgraded by one level if (1) a sample size with adequate power for the outcome was not calculated and reported and (2) the upper or lower 95% CI spanned an effect size of 0.5 in either direction
Publication bias	The presence of publication bias as assessed by funnel plots, where applicable

CI, confidence intervals; GRADE, Grading of Recommendations Assessment, Development and Evaluation; PEDro, Physiotherapy Evidence Database.

In the case of a single trial outcome, we *a priori* graded the evidence as “low certainty,” and if the study had a “high risk” of bias, the evidence was downgraded to “very low certainty” (35, 36).

## 3 Results

### 3.1 Study selection

The results of the study selection process are presented in Figure 1. The initial research identified 480 records. After removing duplicates and screening the title and abstract, 130 articles were sought for retrieval and found to be potentially eligible for review. After reading the full texts of the 130 articles were scrutinized for eligibility based on our inclusion and exclusion criteria. After complete screening, nine studies were included in the final analysis.

### 3.2 Study characteristics and participants

Study characteristics such as sample size, age, outcome measures, and follow-up are presented in Tables 2–5. The nine eligible studies were published between 2007 and 2015 and included 433 participants, of which 342 were males (79%) with a mean age ranging from 24 to 35 years. Men outnumbered women in all studies except for one study, where there were more women than men (37). The participants in all studies were patients admitted to rehabilitation clinics and hospitals (37–45).

**Table 2:** OKC vs. CKC exercises in patients after ACLR surgery.

Study	Sample size	Age	Outcome measures	Follow-up
Chrzan et al. (37)	40 patients were randomized to OKC (*n* = 20) and CKC (*n* = 20)	OKC: 26.2 ± 4.22 CKC: 27.3 ± 8.52	Function: Lysholm score and IKDC form	Baseline and after 2 weeks
Kang et al. (38)	36 patients were randomized to OKC (*n* = 18) and CKC (*n* = 18)	OKC: 29.9 ± 2.3 CKC: 29 ± 4.0	Strength: isokinetic quadriceps. The knee joint moved from 0° to 90° at a speed of 60° /s in four forced repetitions to obtain peak torque	Baseline and 12 weeks (24 weeks post-surgery)
Perry, et al. (39)	49 patients were randomized to OKC (*n* = 24) and CKC (*n* = 25)	OKC: 33 ± 7 CKC: 33 ± 8	Laxity: Knee Signature System arthrometer with the knee in 25° flexion (178 N) Function: Hughston Clinic questionnaire Functional performance: horizontal jump, vertical jump, and cross-over jump.	Baseline (8 weeks) and at 14 weeks
Uçar et al. (40)	66 patients were randomized to CKC (*n* = 28) and OKC (*n* = 30)	CKC: 27.4 ± 10.5 OKC: 28.1 ± 11.9	Pain: subjective pain visual analog scale Function: Lysholm score	Baseline, 3 months, and 6 months

**Table 3:** OKC vs. CKC exercises in patients with ACL-deficient knees.

Study	Sample size	Age	Outcome measures	Follow-up
Perry et al. (41)	64 patients were randomly randomized to OKC (*n* = 32) and CKC (*n* = 32)	OKC: 31 ± 8 CKC: 30 ± 7	Laxity: Knee Signature System arthrometer with the knee in 25° flexion (178 N) Function: Hughston Clinic questionnaire Functional performance: horizontal, vertical, and triple cross-over jump	Baseline (4 weeks) and after 6 weeks (10 weeks)
Tagesson et al. (42)	42 patients were randomized to OKC (*n* = 22) and CKC (*n* = 20)	CKC: 27 (15–44) OKC: 25 (16–41)	Laxity: Lachman 90 N and Lachman 134 N Function: Lysholm score Strength: isokinetic knee extension Functional performance: single-legged vertical jump and single-legged jump for distance	Baseline and after 4 months

**Table 4:** Early vs. late start of OKC exercises in patients after ACLR surgery.

Study	Sample size	Age	Outcome measures	Follow-up
Fukuda et al. (43)	45 patients randomized to early OKC (*n* = 23) and late OKC (*n* = 22)	Hamstrings graft Early OKC: 26.5 ± 8.5 Late OKC: 23.9 ± 5.5	Laxity roll meter device, with the knee in 25° of flexion Strength: isometric quadriceps Function: Lysholm score Functional performance: single-leg hop test and triple cross-over hop Pain: Numeric Pain Rating Scale (NPRS)	At 12 weeks, 19 weeks, 25 weeks, 17 months
Heijne and Werner (44)	68 patients randomized to early start (*n* = 19) and late start (*n* = 15) OKC (patellar graft) and early start (*n* = 17) and late start (hamstring graft)	Early OKC (patellar graft): 31 ± 8 Late OKC (patellar graft) 27 ± 5 Early OKC (hamstrings graft): 30 ± 8 Late OKC (hamstrings graft): 31 ± 9	Laxity: KT-1000 arthrometer, with the knee in 20° of flexion Strength (%): Kin-Com dynamometer (Quadriceps ratio between asymptomatic and reconstructed leg) Pain: modified anterior knee pain score	Pre-op, 3, 5, and 7 months

**Table 5:** The effectiveness of OKC resistance exercises in a patient with ACL-deficient knees.

Study	Sample size	Age	Outcome measures	Follow-up
Barcellona et al. (45)	30 patients randomized to standard protocol (*n* = 13), LOW (standard and OKC) (2 sets, 20 RM) exercises (*n* = 11) and HIGH (standard and OKC) (20 sets, 2 RM) exercises (*n* = 12)	Standard: 35 ± 9 LOW: 32 ± 5 HIGH: 29 ± 7	Laxity: KT-2000 arthrometer, with a force of 133 N at 30° knee flexion (injured minus uninjured knee laxity corrected for lateral hamstring activity) Function: Lysholm score, Tegner score, IKDC Subjective Knee Form, Hughston Clinic, and SF-36 Functional performance: single horizontal hop test for distance	Baseline, at 6 and 12 weeks

The diagnostic criteria in five studies were either based on participants having undergone ACL reconstruction surgery (37–39, 43, 44) or based on clinical testing and magnetic resonance (40), arthroscopic examination or magnetic resonance imaging (42), and arthroscopic examination, magnetic resonance imaging and clinical testing (41, 45).

### 3.3 Intervention characteristics

Intervention characteristics for all studies are presented in Table 6. In these studies, OKC exercises were a common intervention in one of the groups. Six studies (37–44) used two interventions.

**Table 6:** Characteristics of the interventions.

Study	Interventions
Barcellona et al. **(**45)	Group 1: STAND—standard rehabilitation protocol (unilateral OKC resistance exercises for knee extensors on a seated knee extension machine. (Dosage: 1–3 weeks, 3 sets × 20 RM; 4–6 weeks, 3 sets × 6 RM; increase load when pain is less than 5) Group 2: LOW—standard protocol with the addition of seated knee extensor open kinetic chain resistance training at loads of 2 sets of 20 repetition maximum (RM) and 20 sets of 2 RM and low resistance Group 3: HIGH—standard protocol with the addition of seated knee extensor open kinetic chain resistance training at loads of 20 sets of 2 RM All groups: bike for 10 min; hamstrings, quadriceps, iliotibial band, and calf stretching; lunges; patellar, tibiofemoral, and soft tissue mobilization; proprioception, balance, and agility training; isotonic hamstrings strength; step-ups; calf raises; interferential stimulation; ice therapy
Chrzan et al. (37)	Group 1: Steadman Hawkins Clinic Vail (Colorado, USA) program based on CKC exercises [bilateral toe rises, bilateral leg presses, passive knee flexion to 90°, supine isometric quadriceps contractions with heel elevated, seated isometric quadriceps contractions with heel elevated, stepper and walking on a raised beam (balance board)] Group 2: Chester Knee Clinic and Cartilage Repair Centre (England) protocol based on OKC exercises (knee extension with a skipping rope, walking backwards, step-ups, unilateral stance, stair master, unilateral calf raises, lunges, and active heel rises) Both groups: stationary bike, heel props, passive heel rises, heel slides, partial squats and wall slides, and quadriceps exercises. Three series of 50 repetitions of each exercise, gradually increased daily to six series of 50 repetitions.
Fukuda et al. (43)	Group 1: early start OKC (commencing at the fourth postoperative week) (seated knee extension with NMES (isokinetic, 45°–90°; isometric, 60°) Group 2: late start OKC (commencing at the 12th postoperative week) (seated knee extension with NMES (isokinetic, 0°–90°) Both groups: 25-week duration 3 sessions per week; approximately 70 sessions PWB at 2 weeks post-surgery, with 2 crutches Isometric CKC exercises for hip and knee strengthening started in the second postoperative week, followed by dynamic CKC exercises in the sixth postoperative week 1 week: PROM extension/flexion, patellar mobilizations 2 weeks: bike core, strength (hip, calf, squat), leg press, balance 3 weeks: FWB without aid; AROM flexion 5–7 weeks: increase ROM in leg press, bridges, step-ups/step-downs, SL sit-to-stand, trampoline 8–9 weeks: SL HR, increase ROM in leg press, hamstring curl 10–11 weeks: straight-line running, hip strength with Thera-Band, SL trampoline, DL jumping 4 months: SL squats, SL jumping, lateral shuttle runs, seated knee extension 5–6 months: continued plyometric and agility training, pivoting, sport-specific training
Heijne and Werner (44)	Group 1 (P4): patellar graft with an early start of quadriceps CKC exercises Group 2 (P12): patellar graft with a late start of quadriceps CKC exercises Group 3 (H4): hamstring graft with an early start of quadriceps OKC exercises Group 4 (H12): hamstring graft with a late start of quadriceps OKC exercises. **Patellar and hamstring grafts** 4 weeks: knee extension, 90°–40° 5 weeks: knee extension, 90°–20° 6 weeks: knee extension, 90°–0° 7 weeks: knee extension with resistance **Patellar tendon and hamstring grafts** Immediately commence 0°–90° OKC exercises. No resistance for the first week **All groups** 0–2 weeks: patellar mobilizations, PROM extension, AROM flexion/extension, gait, squats, HR 2–5 weeks: bike, leg press, and curl in machine 6–8 weeks: AROM knee extension from 30° to 0°, step-ups/step-downs, SL HR and sit-to-stand, lunges, DL trampoline 9–11 weeks: jumping, SL trampoline, straight-line running 3–4 months: OKC quadriceps full ROM, continue balance and plyometric drills 4–6 months: running and cutting, acceleration/deceleration, sport-specific training
Kang et al. (38)	Group 1: OKC exercises (SLR, leg extensions, leg curls) Group 2: CKC exercises (squats, leg press, Squat, leg press, lunges) 12-week duration 30-min sessions, 3 sessions per week All exercises: 5 sets of 12 repetitions at 70% intensity of 1 RM Stationary bike warm-up/cool-down
Perry et al. (39)	Group 1: unilateral CKC resistance exercises on a leg press machine for hip/knee extensors. (Dosage: 1–3 weeks, 3 sets × 20 RM; 4–6 weeks, 3 sets × 6 RM; increase load when pain is less than 5) Group 2: OKC resistance exercises with ankle weights or knee extension/ham curl machine, Hip extension with ankle weights (Dosage: 1–3 weeks, 3 sets × 20 RM; 4–6 weeks, 3 sets × 6 RM; increase load when pain is less than 5) Both groups: bike for 10 min; hamstrings, quadriceps, iliotibial band, and calf stretching; lunges; patellar, tibiofemoral, and soft tissue mobilization; proprioception, balance, and agility training; isotonic hamstrings strength; step-ups; calf raises; interferential stimulation; ice therapy
Perry et al. (41)	Group 1: unilateral CKC resistance exercises on a leg press machine for hip/knee extensors. (Dosage: 1–3 weeks, 3 sets × 20 RM; 4–6 weeks, 3 sets × 6 RM; increase load when pain is less than 5) Group 2: unilateral OKC resistance exercises with ankle weights or knee extension/ham curl machine, hip extension with ankle weights. (Dosage: 1–3 weeks, 3 sets × 20 RM; 4–6 weeks, 3 sets × 6 RM; increase load when pain is less than 5) Both groups: bike for 10 min; hamstrings, quadriceps, iliotibial band, and calf stretching; lunges; patellar, tibiofemoral, and soft tissue mobilization; proprioception, balance, and agility training; isotonic and ballistic hamstrings strength; step-ups; calf raises; interferential stimulation; ice therapy
Tagesson et al. (42)	Group 1: OKC seated knee extension on one leg was the primary quads strengthening exercise (3 sessions/week for 4 months, 3 sets, 10 reps for each exercise), proprioceptive, strength, and coordination exercises, sport-specific exercises, and functional activities. Group 2: CKC squatting on one leg was the primary quads strength exercise (3 sessions/week for 4 months, 3 sets 10 reps for each exercise), proprioceptive, strength and coordination exercises and sport-specific exercises, and functional activities
Uçar et al. (40)	Group 1: CKC squatting lunges, standing weight shift, wall sits, single-legged quadriceps dips, lateral step-ups Group 2: OKC isometric quadriceps, flexor/extensor bench, isotonic quadriceps, long leg press on/off, knee flexion/extension stretching Both groups: Jones bandage, elevation, and cold pack after operation 24 h post-surgery: encouraged standing and WB with crutches Days 3–7: ankle pumps, isometric quadriceps, SLR Days 7–15: knee PROM with CPM from 0° to 90°, ambulation with crutches (FWB) Days 15–30: if knee flexion is >110°, then allowed to walk quickly, run on a smooth surface, and ascend/descend stairs

1 RM, one-repetition maximum; AROM, active range of movement; CKC, closed kinetic chain; CPM, continuous passive motion; DL, double leg; FWB, full weight-bearing; HR, heel raise; NMES, neuromuscular electrical stimulation; OKC, open kinetic chain; PROM, passive range of movement; PWB, partial weight-bearing; ROM, range of movement; SL, single leg; SLR, straight leg raise; WB, weight-bearing.

### 3.4 Outcome measures

The visual analog scale (40), numerical pain rating scale (43), and modified anterior knee pain score (44) were used to evaluate the outcome measures for analog pain in the studies selected in this systematic review. The three instruments used to measure pain are valid, reliable, and suitable for use in clinical practice (46–48).

For function, the Lysholm knee scoring scale questionnaire (37, 40, 42, 43, 45) was used, alongside the Tegner score (37, 45), the Hughston Clinic knee self-assessment questionnaire (39, 41, 45), and the IKDC form (37). These instruments are valid and reliable (49–51).

For the assessment of muscle strength, the hand dynamometer (43) and the isokinetic dynamometer (38, 42, 44) were used. These dynamometers are safe, valid, and reliable for use in clinical practice (52–55).

To assess anterior knee laxity, the studies included in this systematic review used the KT-2000 arthrometer (45), rolimeter arthrometer (43), KT-1000 (44), and Knee Signature System (39, 41). The four instruments that measured anterior knee laxity are distinguished by their validity and reliability (56–61).

### 3.5 Methodological quality

The risk of bias assessment with the PEDro scale showed that out of the nine included studies, eight (89%) were of low quality, with a mean score of 5.6. The scoring of studies for the risk of bias ranged from 5 to 8 (Table 7). Of the eligible studies, one presented high methodological quality (43), and the remaining eight were of moderate methodological quality (37–42, 44, 45). The main methodological concerns were lack of therapist (9/9), patient blinding (9/9), and intention-to-treat analysis (8/9).

**Table 7:** Study quality ratings.

PEDro scale	Barcellona et al. (45)	Chrzan et al. (37)	Fukuda et al. (43)	Heijne and Werner (44)	Kang et al. (38)	Perry et al. (39)	Perry et al. (41)	Tagesson et al. (42)	Uçar et al. (40)	Percent (%)
Eligibility criteria specified	Yes	Yes	Yes	Yes	Yes	Yes	Yes	Yes	Yes	100%
Random allocation	Yes	Yes	Yes	Yes	Yes	Yes	Yes	Yes	Yes	100%
Concealed allocation	No	No	Yes	Yes	No	No	No	Yes	Yes	44.4%
Baseline comparability	Yes	Yes	Yes	Yes	Yes	Yes	Yes	Yes	Yes	100%
Participant blinding	No	No	No	No	No	No	No	No	No	0%
Therapist blinding	No	No	No	No	No	No	No	No	No	0%
Assessor blinding	Yes	No	Yes	Yes	No	Yes	Yes	No	No	55.5%
Adequate follow-up	No	Yes	Yes	No	No	No	No	Yes	Yes	44.4%
Intention-to-treat analysis	No	No	Yes	No	No	No	No	No	No	11.1%
Between-group comparisons	Yes	Yes	Yes	Yes	Yes	Yes	Yes	Yes	Yes	100%
Point estimates variability	Yes	Yes	Yes	Yes	Yes	Yes	Yes	Yes	Yes	100%
Total PEDro score (Risk of bias)	5/10 (High risk)	5/10 (High risk)	8/10 (Low risk)	6/10 (High risk)	4/10 (High risk)	5/10 (High risk)	5/10 (High risk)	6/10 (High risk)	6/10 (High risk)	

### 3.6 Effects of interventions

#### 3.6.1 OKC vs. CKC exercises in patients after ACLR

Four RCTs examined the effects of OKC vs. CKC exercises in patients after ACLR (37–40), of which all were at high risk of bias (Table 7).

##### 3.6.1.1 Function

Three studies (37, 39, 40) evaluated knee function using Lysholm and International Knee Documentation Committee (IKDC) Subjective Knee Form scores or Hughston knee score (Table 4). Based on very low certainty evidence, there was a significant difference in Lysholm score in favor of CKC compared to OKC exercises at 2 weeks (MD = −8.45) (37) and at 3 months of follow-up (MD = −2.3) (40). Finally, based on very low certainty of evidence, there was a significant difference in Lysholm score in favor of CKC compared to OKC exercises at 6 months (MD = −9.8) (40). There was very low certainty evidence of a significant difference in IKDC form in favor of OKC compared to CKC exercises at 2 weeks of follow-up (MD = 8.45) (37) and very low certainty evidence of a non-significant difference in Hughston knee score between OKC and CKC exercises at 14 weeks of follow-up (MD = −3.0) (Table 8).

**Table 8:** Summary of evidence for the effects of OKC vs. CKC exercises after ACLR surgery.

Outcome measure	Comparisons		Relative effect [95% CI]	CKC/OKC (*n* studies)	Quality of evidence (GRADE)	Evidence and significance
	Average estimate in the OKC group	Average estimate in the CKC group				
Lysholm score 2 weeks	OKC: Mean ± SD was 69.75 ± 13.81	CKC: Mean ± SD was 78.2 ± 10.87	MD −8.45 [−16.15, −0.75] Statistically significant difference	20/20 (1)	⊕◯◯◯ Very low^a^	Very low certainty evidence of a significant difference in Lysholm score in favor of CKC compared to OKC at 2 weeks.
Lysholm score 3 months	OKC: Mean ± SD was 78.5 ± 14.5	CKC: Mean ± SD was 80.8 ± 19.1	MD −2.3 [−10.99, 6.39] Non-statistically significant difference	28/30 (1)	⊕◯◯◯ Very low^a^	Very low certainty evidence of a non-significant difference in Lysholm score between OKC and OKC at 3 months of follow-up.
Lysholm score 6 months	OKC: Mean ± SD was 84.3 ± 9.1	CKC: Mean ± SD was 94.1 ± 8.5	MD −9.8 [−14.34, −5.26] Statistically significant difference	28/30 (1)	⊕◯◯◯ Very low1	Very low certainty evidence of a significant difference in Lysholm score in favor of CKC compared to OKC at 6 months.
IKDC form 2 weeks	OKC: Mean ± SD was 78.20 ± 10.87	CKC: Mean ± SD was 69.75 ± 13.81	MD 8.45 [0.75, 16.15] Statistically significant difference	20/20 (1)	⊕◯◯◯ Very low^a^	Very low certainty evidence of a significant difference in IKDC form in favor of OKC compared to CKC at 2 weeks of follow-up.
Quadriceps isokinetic strength 12 weeks	OKC: Mean ± SD was 161.1 ± 40.1	CKC: Mean ± SD was 133 ± 36.1	MD 28.1 [3.17, 53.03] Statistically significant difference	18/18 (1)	⊕◯◯◯ Very low^a^	Very low certainty evidence of a significant difference in quadriceps isokinetic strength in favor of OKC compared to CKC at 12 weeks.
Laxity 14 weeks	OKC: Mean ± SD was 12 ± 3	CKC: Mean ± SD was 12 ± 3	MD 0 [−1.72, 1.72] Non-statistically significant difference	23/24 (1)	⊕◯◯◯ Very low^a^	Very low certainty evidence of a non-significant difference in laxity between OKC and CKC at 14 weeks of follow-up.
Hughston knee score 14 weeks	OKC: Mean ± SD was 29 ± 13	CKC: Mean ± SD was 12 ± 13	MD −3.0 [−10.28, 4.28] Non-statistically significant difference	24/25 (1)	⊕◯◯◯ Very low^a^	Very low certainty evidence of a non-significant difference in Hughston knee score between OKC and CKC at 14 weeks of follow-up.
Unilateral horizontal hop 14 weeks	OKC: Mean ± SD was 0.77 ± 0.17	CKC: Mean ± SD was 0.74 ± 0.15	MD 0.03 [−0.09, 0.15] Non-statistically significant difference	14/15 (1)	⊕◯◯◯ Very low^a^	Very low certainty evidence of a non-significant difference in unilateral horizontal hop between OKC and CKC at 14 weeks of follow-up.
Unilateral vertical hop 14 weeks	OKC: Mean ± SD was 0.75 ± 0.15	CKC: Mean ± SD was 0.78 ± 0.11	MD −0.03 [−0.12, 0.06] Non-statistically significant difference	15/15 (1)	⊕◯◯◯ Very low^a^	Very low certainty evidence of a non-significant difference in unilateral vertical hop between OKC and CKC at 14 weeks of follow-up.
Triple cross-over hop 14 weeks	OKC: Mean ± SD was 0.79 ± 0.15	CKC: Mean ± SD was 0.81 ± 0.26	MD −0.02 [−0.23, 0.19] Non-statistically significant difference	9/8 (1)	⊕◯◯◯ Very low^a^	Very low certainty evidence of a non-significant difference in unilateral triple cross-over hop between OKC and CKC at 14 weeks of follow-up.
Pain (VAS) 3 months	OKC: Mean ± SD was 48.6 ± 11.4	CKC: Mean ± SD was 41.1 ± 12.9	MD 7.50 [1.24, 13.76] Statistically significant difference	20/20 (1)	⊕◯◯◯ Very low^a^	Very low certainty evidence of a significant difference in pain in favor of CKC compared to OKC at 3 months.
Pain (VAS) 6 months	OKC: Mean ± SD was 27.2 ± 9.9	CKC: Mean ± SD was 22.1 ± 10.5	MD 5.10 [−0.15, 10.35] Non-statistically significant difference	28/30 (1)	⊕◯◯◯ Very low^a^	Very low certainty evidence of a non-significant difference in pain between OKC and CKC at 14 weeks of follow-up.

GRADE, Grading of Recommendations Assessment, Development and Evaluation; CKC, close kinetic chain; OKC, open kinetic chain; MD, mean difference; SD, standard deviation.

^a^ Downgraded due to risk of bias.

^b^ Downgraded due to inconsistency.

^c^ Downgraded due to indirectness.

^d^ Downgraded due to imprecision.

##### 3.6.1.2 Laxity

Only one study (39) evaluated the effect of OKC and CKC exercises on knee laxity at 14 weeks of follow-up. This was based on very low certainty evidence of a non-significant difference between the comparators (MD = 0) (Table 8).

##### 3.6.1.3 Strength

Only one study (38) evaluated the effects on quadriceps isokinetic strength. There was very low certainty evidence of a significant difference in quadriceps isokinetic strength in favor of OKC compared to CKC exercises at 24 weeks (MD = 28.1) (Table 8).

##### 3.6.1.4 Functional performance

Only one study (39) evaluated the effects of unilateral horizontal hop (MD = 0.03), unilateral vertical hop (MD = −0.03), and triple cross-over hop (MD = −0.02). There was very low certainty evidence of a non-significant difference in all three tests between OKC and CKC exercises at 14 weeks of follow-up (Table 8).

##### 3.6.1.5 Pain

One study (40) evaluated the effects on pain using the subjective pain visual analog scale at 3 and 6 months. Based on very low certainty evidence, there was a significant difference in pain in favor of CKC compared to OKC exercises at 3 months (MD = 7.50). However, there was very low certainty evidence of a non-significant difference in pain intensity between the two exercises at 14 weeks of follow-up (MD = 5.10) (Table 8).

#### 3.6.2 OKC vs. CKC exercises in patients with ACL deficiency

Two studies (41, 42) evaluated the effects of OKC and CKC exercises on knee laxity and function in patients with ACL-deficient knees. Both were at high risk of bias (Table 7).

##### 3.6.2.1 Laxity

Based on very low certainty evidence, there was a significant difference in laxity using the Knee Signature System (mm) 178N with the knee in 25° flexion (178N) in favor of OKC compared to CKC exercises at 10 weeks (MD = −2.45) (41) and using the Lachman test 90N (MD = −0.40) at 4 weeks. On the other hand, there was very low certainty evidence of a non-significant difference in laxity using the Lachman test 134N (MD = −0.10) at 4 months of follow-up (41) (Table 9).

**Table 9:** Summary of evidence for the effects of OKC vs. CKC exercises in patients with ACL-deficient knees.

Outcome measure	Comparisons		Relative effect [95% CI]	CKC/OKC (*n* studies)	Quality of evidence (GRADE)	Evidence and significance
	Average estimate in the OKC group	Average estimate in the CKC group				
Laxity [Knee Signature System (mm)] 178 N 10 weeks	OKC: Mean ± SD was 13 ± 3	CKC: Mean ± SD was 15 ± 3	MD −2.45 [−3.54, −0.46] Statistically significant difference	29/29 (1)	⊕◯◯◯ Very low^a^	Very low certainty evidence of a significant difference in laxity in favor of OKC compared to CKC at 10 weeks.
Laxity (Lachman 90 N) 4 months	OKC: Mean ± SD was 5.9 ± 1.5	CKC: Mean ± SD was 6.3 ± 2.1	MD −0.40 [−1.51, −0.71] Statistically significant difference	22/20 (1)	⊕◯◯◯ Very low^a^	Very low certainty evidence of a significant difference in laxity (90 N) in favor of OKC compared to CKC at 4 months of follow-up.
Laxity (Lachman 134 N) 4 months	OKC: Mean ± SD was 7.7 ± 1.5	CKC: Mean ± SD was 7.8 ± 2.5	MD −0.10 [−1.31, 1.12] Non-statistically significant difference	22/22 (1)	⊕◯◯◯ Very low^a^	Very low certainty evidence of a non-significant difference in laxity (134 N) between OKC and CKC at 4 months of follow-up.
Isokinetic quadriceps strength 4 months	OKC: Mean ± SD was 96 ± 14	CKC: Mean ± SD was 84 ± 15	MD 12 [3.2, 20.80] Statistically significant difference	22/20 (1)	⊕◯◯◯ Very low^a^	Very low certainty evidence of a significant difference in isokinetic quadriceps strength in favor of OKC compared to CKC at 4 months.
Hughston knee score 10 weeks	OKC: Mean ± SD was 0.33 ± 0.18	CKC: Mean ± SD was 0.29 ± .15	MD 0.04 [−0.05, 0.13] Non-statistically significant difference	29/29 (1)	⊕◯◯◯ Very low^a^	Very low certainty evidence of a non-significant difference in Hughston knee score between OKC and CKC at 10 weeks of follow-up.
Horizontal hop test (inj/uninj) 10 weeks	OKC: Mean ± SD was 0.88 ± 0.08	CKC: Mean ± SD was 0.88 ± .0.17	MD 0.00 [−0.07, 0.07] Non-statistically significant difference	25/27 (1)	⊕◯◯◯ Very low^a^	Very low certainty evidence of a non-significant difference in horizontal hop test between OKC and CKC at 10 weeks of follow-up.
Vertical hop test (inj/uinj) 10 weeks	OKC: Mean ± SD was 0.75 ± 0.15	CKC: Mean ± SD was 0.78 ± .0.11	MD −0.03 [−0.12, 0.06] Non-statistically significant difference	15/15 (1)	⊕◯◯◯ Very low^a^	Very low certainty evidence of a non-significant difference in vertical hop test between OKC and CKC at 10 weeks of follow-up.
Triple cross-over hop test (inj/uninj) 10 weeks	OKC: Mean ± SD was 0.79 ± 0.15	CKC: Mean ± SD was 0.81 ± .0.26	MD −0.02 [−0.23, 0.19] Non-statistically significant difference	9/18 (1)	⊕◯◯◯ Very low^a^	Very low certainty evidence of a non-significant difference in triple cross-over test between OKC and CKC at 10 weeks of follow-up.
Lysholm score 4 months	OKC: Mean ± SD was 90.59 ± 9.16	CKC: Mean ± SD was 87.95 ± .12.71	MD 2.64 [−4.12, 9.40] Non-statistically significant difference	22/20 (1)	⊕◯◯◯ Very low^a^	Very low certainty evidence of a non-significant difference in Lysholm score between OKC and CKC at 4 months of follow-up.
Single-legged vertical jump test 4 months	OKC: Mean ± SD was 96 ± 8	CKC: Mean ± SD was 93 ± .15	MD 3.00 [−4.77, 10.77] Non-statistically significant difference	20/18 (1)	⊕◯◯◯ Very low^a^	Very low certainty evidence of a non-significant difference in single-legged vertical jump test between OKC and CKC at 4 months of follow-up.
Single-legged jump for distance test 4 months	OKC: Mean ± SD was 94 ± 15	CKC: Mean ± SD was 91 ± .11	MD 3.00 [−5.59, 11.59] Non-statistically significant difference	18/18 (1)	⊕◯◯◯ Very low^a^	Very low certainty evidence of a non-significant difference in a single-legged jump for distance test between OKC and CKC at 4 months of follow-up.

GRADE, Grading of Recommendations Assessment, Development and Evaluation; CKC, close kinetic chain; OKC, open kinetic chain; MD, mean difference; SD, standard deviation.

^a^ Downgraded due to risk of bias.

^b^ Downgraded due to inconsistency.

^c^ Downgraded due to indirectness.

^d^ Downgraded due to imprecision.

##### 3.6.2.2 Strength

Only one study (42) evaluated the effects of isokinetic quadriceps strength. Based on very low certainty evidence, there was a significant difference in isokinetic quadriceps strength in favor of OKC compared to CKC exercises at 4 months (MD = 12) (Table 9).

##### 3.6.2.3 Function

Two studies (41, 42) evaluated the effect on function using the Hughston Clinic questionnaire or the Lysholm score (Table 5). There was very low certainty evidence of a non-significant difference in Hughston knee score between OKC and CKC exercises at 10 weeks of follow-up (MD = 0.04) (41) and in Lysholm score at 4 months of follow-up (MD = 2.64) (Table 9).

##### 3.6.2.4 Functional performance

There was very low certainty evidence of a non-significant difference in the horizontal hop test (MD = 0.00), in the vertical hop test (MD −0.03), and in the triple cross-over hop test (MD = −0.02) between OKC and CKC exercises at 10 weeks of follow-up (41). In addition, there was very low certainty evidence of a non-significant difference in single-legged vertical jump test (MD = 3.00) and single-legged jump for distance test (MD = 3.00) between OKC and CKC exercises at 4 months of follow-up (42) (Table 9).

#### 3.6.3 Early vs. late start of OKC exercises in patients after ACLR

##### 3.6.3.1 Laxity

Two studies (43, 44) evaluated anterior tibial laxity as the difference in anterior knee laxity between the healthy and the ACL reconstructed site using arthrometry at different knee angles, of which only one was of low risk of bias (43) (Table 7).

###### 3.6.3.1.1 Short term

A very low certainty evidence of a non-significant difference in patellar graft laxity between CKC and OKC exercises exists (MD = 0.10) (44). Pooled results from both studies (43, 44) suggested a very low certainty evidence of a non-significant difference in hamstring graft laxity between the two techniques (SMD = −0.45) (Table 10).

**Table 10:** Summary of evidence for the effects of early vs. late OKC exercises after ACLR surgery.

Outcome measure	Comparisons		Relative effect [95% CI]	CKC/OKC (*n* studies)	Quality of evidence (GRADE)	Evidence and significance
	Average estimate in the early OKC group	Average estimate the in late OKC group				
Laxity- Patellar graft Short term	Early OKC: Mean ± SD was 1.2 ± 1.42	Late OKC: Mean ± SD was 1.1 ± 1.48	MD 0.10 [−0.89, 1.09] Non-statistically significant difference	15/18 (1)	⊕◯◯◯ Very low^a^	Very low certainty evidence of a non-significant difference in laxity of patellar graft between CKC and OKC at short-term follow-up.
Laxity-Hamstring graft Short term	Early OKC: Pooled weighted mean ± SD was 1.74 ± 1.45 (mean range 0.9–2.6)	Late OKC: Pooled weighted mean ± SD was 2.51 ± 0.50 (mean range 2.3–2.7)	SMD −0.45 [−1.25, 0.35] Non-statistically significant difference	34/35 (2)	⊕◯◯◯ Very low^a,d^	Very low certainty evidence of a non-significant difference in hamstring graft laxity between CKC and OKC at short-term follow-up.
Laxity- Patellar graft Medium-term	Early OKC: Mean ± SD was 1.3 ± 1.43	Late OKC: Mean ± SD was 1.6 ± 1.57	MD −0.30 [−1.42, 0.82] Non-statistically significant difference	12/16 (1)	⊕◯◯◯ Very low^a^	Very low certainty evidence of a non-significant difference in laxity of patellar graft between CKC and OKC at medium-term follow-up.
Laxity- Hamstring graft Medium-term	Early OKC: Pooled weighted mean ± SD was 2.07 ± 0.52 (mean range 1.3–2.7)	Late OKC: Pooled weighted mean ± SD was 2.75 ± 0.36 (mean range 2.5–2.9)	SMD −0.42 [−1.08, 0.23] Non-statistically significant difference	31/32 (2)	⊕◯◯◯ Very low^a,d^	Very low certainty evidence of a non-significant difference in hamstring graft laxity between CKC and OKC at medium-term follow-up.
Laxity- Patellar graft Long term	Early OKC: Mean ± SD was 1.3 ± 1.39	Late OKC: Mean ± SD was 1.3 ± 1.57	MD 0.00 [−1.0,6 1.06] Non-statistically significant difference	14/16 (1)	⊕◯◯◯ Very low^a^	Very low certainty evidence of a non-significant difference in laxity of patellar graft between CKC and OKC at long-term follow-up.
Laxity- Hamstring graft Long term	Early OKC: Pooled weighted mean ± SD was 2.22 ± 0.72 (mean range 1.2–3.0)	Late OKC: Pooled weighted mean ± SD was 2.71 ± 0.38 (mean range 2.3–3.0)	SMD −0.32 [−1.00, 0.37] Non-statistically significant difference	30/31 (2)	⊕◯◯◯ Very low^a,b,d^	Very low certainty evidence of a non-significant difference in hamstring graft laxity between CKC and OKC at long-term follow-up.
Laxity- Hamstring graft Very long term	Early OKC: Mean ± SD was 2.7 ± 1.4	Late OKC: Mean ± SD was 2.7 ± 1.4	MD 0.00 [−0.93, 0.93] Non-statistically significant difference	18/17 (1)	⊕⊕◯◯ Low^a^	Low certainty evidence of a non-significant difference in hamstring graft laxity between CKC and OKC at very long-term follow-up.
Isometric quadriceps strength Short term	Early OKC: Mean ± SD was 81.2 ± 11.0	Late OKC: Mean ± SD was 81.6 ± 17.3	MD −0.40 [−10.07, 9.27] Non-statistically significant difference	18/17 (1)	⊕⊕◯◯ Low^a^	Low certainty evidence of a non-significant difference in isometric quadriceps strength between early OKC and late OKC at 12 weeks.
Isometric quadriceps strength Medium-term	Early OKC: Mean ± SD was 91.8 ± 11.9	Late OKC: Mean ± SD was 87.0 ± 13.5	MD 4.80 [−3.65, 13.25] Non-statistically significant difference	18/17 (1)	⊕⊕◯◯ Low^a^	Low certainty evidence of a non-significant difference in isometric quadriceps strength between early OKC and late OKC at 19 weeks.
Isometric quadriceps strength Long term	Early OKC: Mean ± SD was 94.1 ± 12	Late OKC: Mean ± SD was 89.5 ± 10.7	MD 4.60 [−2.92, 12.12] Non-statistically significant difference	18/17 (1)	⊕⊕◯◯ Low^a^	Low certainty evidence of a non-significant difference in isometric quadriceps strength between early OKC and late OKC at 26 weeks.
Isometric quadriceps strength Very long term	Early OKC: Mean ± SD was 99.7 ± 7.2	Late OKC: Mean ± SD was 95.1 ± 11.8	MD 0.40 [−1.92, 11.12] Non-statistically significant difference	18/17 (1)	⊕⊕◯◯ Low^a^	Low certainty evidence of a non-significant difference in isometric quadriceps strength between early OKC and late OKC at 17 months
Pain (NPRS) Short term	Early OKC: Mean ± SD was 1.1 ± 1	Late OKC: Mean ± SD was 2.6 ± 1.9	MD −1.50 [−2.50, 0.5] Statistically significant difference	18/17 (1)	⊕⊕◯◯ Low^a^	Low certainty evidence of a significant difference in pain in favor of early OKC compared to late OKC at 12 weeks.
Pain (NPRS) Medium-term	Early OKC: Mean ± SD was 0.6 ± 1.2	Late OKC: Mean ± SD was 2.6 ± 7.9	MD −2.10 [−3.16, 1.04] Statistically significant difference	18/17 (1)	⊕⊕◯◯ Low^a^	Low certainty evidence of a significant difference in pain in favor of early OKC compared to late OKC at 19 weeks.
Pain (NPRS) Long term	Early OKC: Mean ± SD was 0.4 ± 0.8	Late OKC: Mean ± SD was 3.0 ± 1.7	MD −2.60 [−3.49, −1.71] Statistically significant difference	18/17 (1)	⊕⊕◯◯ Low^a^	Low certainty evidence of a significant difference in pain in favor of early OKC compared to late OKC at 25 weeks.
Pain (NPRS) Very long term	Early OKC: Mean ± SD was 0.4 ± 1.2	Late OKC: Mean ± SD was 3.5 ± 1.8	MD −3.10 [−4.12, −2.08] Statistically significant difference	18/17 (1)	⊕⊕◯◯ Low^a^	Low certainty evidence of a significant difference in pain in favor of early OKC compared to late OKC at 17 months.
Lysholm score Short term	Early OKC: Mean ± SD was 88.3 ± 7.6	Late OKC: Mean ± SD was 89.3 ± 9.0	MD −1.00 [−6.53, 4.53] Non-statistically significant difference	18/17 (1)	⊕⊕◯◯ Low^a^	Low certainty evidence of a non-significant difference in Lysholm score between early OKC and late OKC at 12 weeks.
Lysholm score Medium-term	Early OKC: Mean ± SD was 95.5 ± 5.1	Late OKC: Mean ± SD was 94.9 ± 4.6	MD 0.60 [−2.61, 3.81] Non-statistically significant difference	18/17 (1)	⊕⊕◯◯ Low^a^	Low certainty evidence of a non-significant difference in Lysholm score between early OKC and late OKC at 19 weeks.
Lysholm score Long term	Early OKC: Mean ± SD was 95.8 ± 4.9	Late OKC: Mean ± SD was 94.3 ± 12.4	MD 1.50 [−4.81, 7.81] Non-statistically significant difference	18/17 (1)	⊕⊕◯◯ Low^a^	Low certainty evidence of a non-significant difference in Lysholm score between early OKC and late OKC at 25 weeks.
Lysholm score Very long term	Early OKC: Mean ± SD was 96.5 ± 4.7	Late OKC: Mean ± SD was 99 ± 4.8	MD −2.50 [−5.65, 0.65] Non-statistically significant difference	18/17 (1)	⊕⊕◯◯ Low^a^	Low certainty evidence of a non-significant difference in Lysholm score between early OKC and late OKC at 17 months.
Single-legged hop test Short term	Early OKC: Mean ± SD was 2.7 ± 1.8	Late OKC: Mean ± SD was 2.6 ± 1.9	MD 0.10 [−1.13, 1.33] Non-statistically significant difference	18/17 (1)	⊕⊕◯◯ Low^a^	Low certainty evidence of a non-significant difference in single-legged hop test between early OKC and late OKC at 12 weeks.
Single-legged hop test Medium-term	Early OKC: Mean ± SD was 2.9 ± 1.4	Late OKC: Mean ± SD was 2.7 ± 1.9	MD 0.20 [−0.91, 1.31] Non-statistically significant difference	18/17 (1)	⊕⊕◯◯ Low^a^	Low certainty evidence of a non-significant difference in single-legged hop test between early OKC and late OKC at 19 weeks.
Single-legged hop test Long term	Early OKC: Mean ± SD was 3 ± 1.5	Late OKC: Mean ± SD was 3 ± 1.7	MD 0.00 [−1.06, 1.06] Non-statistically significant difference	18/17 (1)	⊕⊕◯◯ Low^a^	Low certainty evidence of a non-significant difference in single-legged hop test between early OKC and late OKC at 25 weeks.
Single-legged hop test Very long term	Early OKC: Mean ± SD was 2.7 ± 1.4	Late OKC: Mean ± SD was 2.7 ± 1.4	MD 0.00 [−0.93, 0.93] Non-statistically significant difference	18/17 (1)	⊕⊕◯◯ Low^a^	Low certainty evidence of a non-significant difference in single-legged hop test between early OKC and late OKC at 17 months.
Cross-over hop test Short term	Early OKC: Mean ± SD was 84.5 ± 13.3	Late OKC: Mean ± SD was 81.9 ± 12.2	MD 2.60 [−5.85, 11.05] Non-statistically significant difference	18/17 (1)	⊕⊕◯◯ Low^a^	Low certainty evidence of a non-significant difference in cross-over hop test between early OKC and late OKC at 12 weeks.
Cross-over hop test Medium-term	Early OKC: Mean ± SD was 90.4 ± 8.9	Late OKC: Mean ± SD was 87 ± 11	MD 3.40 [−3.25, 10.05] Non-statistically significant difference	18/17 (1)	⊕⊕◯◯ Low^a^	Low certainty evidence of a non-significant difference in cross-over hop test between early OKC and late OKC at 19 weeks.
Cross-over hop test Long term	Early OKC: Mean ± SD was 94 ± 6.4	Late OKC: Mean ± SD was 92.5 ± 7.6	MD 1.50 [−3.17, 6.17] Non-statistically significant difference	18/17 (1)	⊕⊕◯◯ Low^a^	Very low certainty evidence of a non-significant difference in cross-over hop test between early OKC and late OKC at 25 weeks.
Cross-over hop test Very long term	Early OKC: Mean ± SD was 98.8 ± 6.5	Late OKC: Mean ± SD was 96.2 ± 8.4	MD 2.60 [−2.40, 7.60] Non-statistically significant difference	18/17 (1)	⊕⊕◯◯ Low^a^	Very low certainty evidence of a non-significant difference in cross-over hop test between early OKC and late OKC at 17 months.
Quadriceps strength ratio (patellar graft) Short term	Early OKC: Mean ± SD was 0.55 ± 0.16	Late OKC: Mean ± SD was 0.62 ± 0.17	MD −0.06 [−0.17, 0.05] Non-statistically significant difference	18/15 (1)	⊕◯◯◯ Very low^a^	Very low certainty evidence of a non-significant difference in quadriceps strength between early OKC and late OKC at 3 months.
Quadriceps strength ratio (patellar graft) Medium-term	Early OKC: Mean ± SD was 0.64 ± 0.15	Late OKC: Mean ± SD was 0.7 ± 0.14	MD −0.07 [−0.18, 0.04] Non-statistically significant difference	16/12 (1)	⊕◯◯◯ Very low^a^	Very low certainty evidence of a non-significant difference in quadriceps strength between early OKC and late OKC at 5 months.
Quadriceps strength ratio (patellar graft) Long term	Early OKC: Mean ± SD was 0.75 ± 0.15	Late OKC: Mean ± SD was 0.78 ± 0.14	MD −0.03 [−0.13, 0.07] Non-statistically significant difference	16/14 (1)	⊕◯◯◯ Very low^a^	Very low certainty evidence of a non-significant difference in quadriceps strength between early OKC and late OKC at 7 months.
Quadriceps strength ratio (hamstring graft) Short term	Early OKC: Mean ± SD was 0.72 ± 0.17	Late OKC: Mean ± SD was 0.78 ± 0.17	MD −0.06 [−0.18, 0.06] Non-statistically significant difference	15/17 (1)	⊕◯◯◯ Very low^a^	Very low certainty evidence of a non-significant difference in quadriceps strength between early OKC and late OKC at 3 months.
Quadriceps strength ratio (hamstring graft) Medium-term	Early OKC: Mean ± SD was 0.82 ± 0.17	Late OKC: Mean ± SD was 0.8 ± 0.14	MD 0.02 [−0.10, 0.14] Non-statistically significant difference	12/14 (1)	⊕◯◯◯ Very low^a^	Very low certainty evidence of a non-significant difference in quadriceps strength between early OKC and late OKC at 5 months.
Quadriceps strength ratio (hamstring graft) Long term	Early OKC: Mean ± SD was 0.84 ± 0.15	Late OKC: Mean ± SD was 0.8 ± 0.15	MD 0.04 [−0.07, 0.15] Non-statistically significant difference	14/13 (1)	⊕◯◯◯ Very low^a^	Very low certainty evidence of a non-significant difference in quadriceps strength between early OKC and late OKC at 7 months.

GRADE, Grading of Recommendations Assessment, Development and Evaluation; CKC, close kinetic chain; OKC, open kinetic chain; MD, mean difference; SD, standard deviation; SMD, standardized mean difference.

^a^ Downgraded due to risk of bias.

^b^ Downgraded due to inconsistency.

^c^ Downgraded due to indirectness.

^d^ Downgraded due to imprecision.

###### 3.6.3.1.2 Medium term

A very low certainty evidence of a non-significant difference in patellar graft laxity between CKC and OKC exercises exists (MD = −0.30) (44). Pooled results from both studies (43, 44) suggested a very low certainty evidence of no significant difference in hamstring graft laxity between the two techniques (SMD = 0.42) (Table 10).

###### 3.6.3.1.3 Long term

A very low certainty evidence of a non-significant difference in patellar graft laxity between CKC and OKC exercises exists (MD = 0.00) (44). Pooled results from both studies (43, 44) suggested very low certainty evidence of no significant difference in hamstring graft laxity between the two techniques (SMD = −0.32) (Table 10).

###### 3.6.3.1.4 Very long term

A low certainty evidence of a non-significant difference in hamstring graft laxity between CKC and OKC exercises exists (MD = 0.00) (43) (Table 10).

##### 3.6.3.2 Strength

Quadriceps strength was measured using a handheld dynamometer (43) and an isokinetic dynamometer (44). Isometric strength (43) and quadriceps muscle strength ratio (reconstructed knee/asymptomatic knee) (44) were the outcome measures.

###### 3.6.3.2.1 Short term

A low certainty evidence of a non-significant difference in isometric quadriceps strength between early and late OKC exercises exists at 12 weeks (MD = 0.40). A very low certainty evidence of a non-significant difference in quadriceps muscle ratio (patellar graft) between early and late OKC exercises exists at 3 months (MD = −0.06). A very low certainty evidence of a non-significant difference in quadriceps muscle ratio (hamstrings graft) between early and late OKC exercises exists at 3 months exists (MD = −0.06) (Table 10).

###### 3.6.3.2.2 Medium term

A low certainty evidence of a non-significant difference in isometric quadriceps strength between early and late OKC exercises exists at 19 weeks (MD = 4.80). A very low certainty evidence of a non-significant difference in quadriceps muscle ratio (patellar graft) between early and late OKC exercises exists at 5 months (MD = −0.07). A very low certainty evidence of a non-significant difference in quadriceps muscle ratio (hamstrings graft) between early and late OKC exists at 5 months (MD = 0.02) (Table 10).

###### 3.6.3.2.3 Long term

A low certainty evidence of a non-significant difference in isometric quadriceps strength between early and late OKC exercises exists at 26 weeks (MD = 4.60). A very low certainty evidence of a non-significant difference in quadriceps muscle ratio (patellar graft) between early and late OKC exists at 7 months (MD = −0.03). A very low certainty evidence of a non-significant difference in quadriceps muscle ratio (hamstrings graft) between early and late OKC exists at 7 months (MD = 0.04) (Table 10).

###### 3.6.3.2.4 Very long term

A low certainty evidence of a non-significant difference in isometric quadriceps strength between early and late OKC exists at 17 months (MD = 0.40) (Table 10).

##### 3.6.3.3 Function

One study (43) assessed patient-reported function using the Lysholm score questionnaire.

###### 3.6.3.3.1 Short term

A low certainty evidence of a non-significant difference in Lysholm score in favor of early compared to late OKC exercises exists at 12 weeks (MD = −1.00) (Table 10).

###### 3.6.3.3.2 Medium term

A low certainty evidence of a non-significant difference in Lysholm score between early and late OKC exercises exists at 19 weeks (MD = 0.60) (Table 10).

###### 3.6.3.3.3 Long term

A low certainty evidence of a non-significant difference in Lysholm score between early and late OKC exercises exists at 25 weeks (MD = 1.50) (Table 10).

###### 3.6.3.3.4 Very long term

A low certainty evidence of a non-significant difference in Lysholm score between early and late OKC exercises exists at 17 months (MD = 2.50) (Table 10).

##### 3.6.3.4 Functional performance

Only one study used the single-legged and cross-over hop tests to assess functional performance (43).

###### 3.6.3.4.1 Short term

A low certainty evidence of a non-significant difference in the single-legged hop test (MD = 0.10) and cross-over hop test (MD = 2.60) between early and late OKC exercises exists at 12 weeks (Table 10).

###### 3.6.3.4.2 Medium term

A low certainty evidence of a non-significant difference in single-legged hop test (MD = 0.20) and cross-over hop test (MD = 3.40) between early and late OKC exercises exists at 19 weeks (Table 10).

###### 3.6.3.4.3 Long term

A low certainty evidence of a non-significant difference in the single-legged hop test (MD = 0.00) and cross-over hop test (MD = 1.50) between early and late OKC exercises exists at 25 weeks (Table 10).

###### 3.6.3.4.4 Very long term

A low certainty evidence of a non-significant difference in the single-legged hop test (MD = 0.00) and cross-over hop test (MD = 2.60) between early and late OKC exercises exists at 17 months (Table 10).

##### 3.6.3.5 Pain

One study (43) assessed pain using the numeric pain rating scale (NPRS).

###### 3.6.3.5.1 Short term

A low certainty evidence of a significant difference in pain in favor of early compared to late OKC exercises at 12 weeks exists (MD = −1.50) (Table 10).

###### 3.6.3.5.2 Medium term

A low certainty evidence of a significant difference in pain in favor of early compared to late OKC exercises exists at 19 weeks (MD = −2.10) (Table 10).

###### 3.6.3.5.3 Long term

A low certainty evidence of a significant difference in pain in favor of early compared to late OKC exercises exists at 25 weeks (MD = −2.60) (Table 10).

###### 3.6.3.5.4 Very long term

A low certainty evidence of a significant difference in pain in favor of early compared to late OKC exercises exists at 17 months (MD = −3.10) (Table 10).

**Figure 2:**
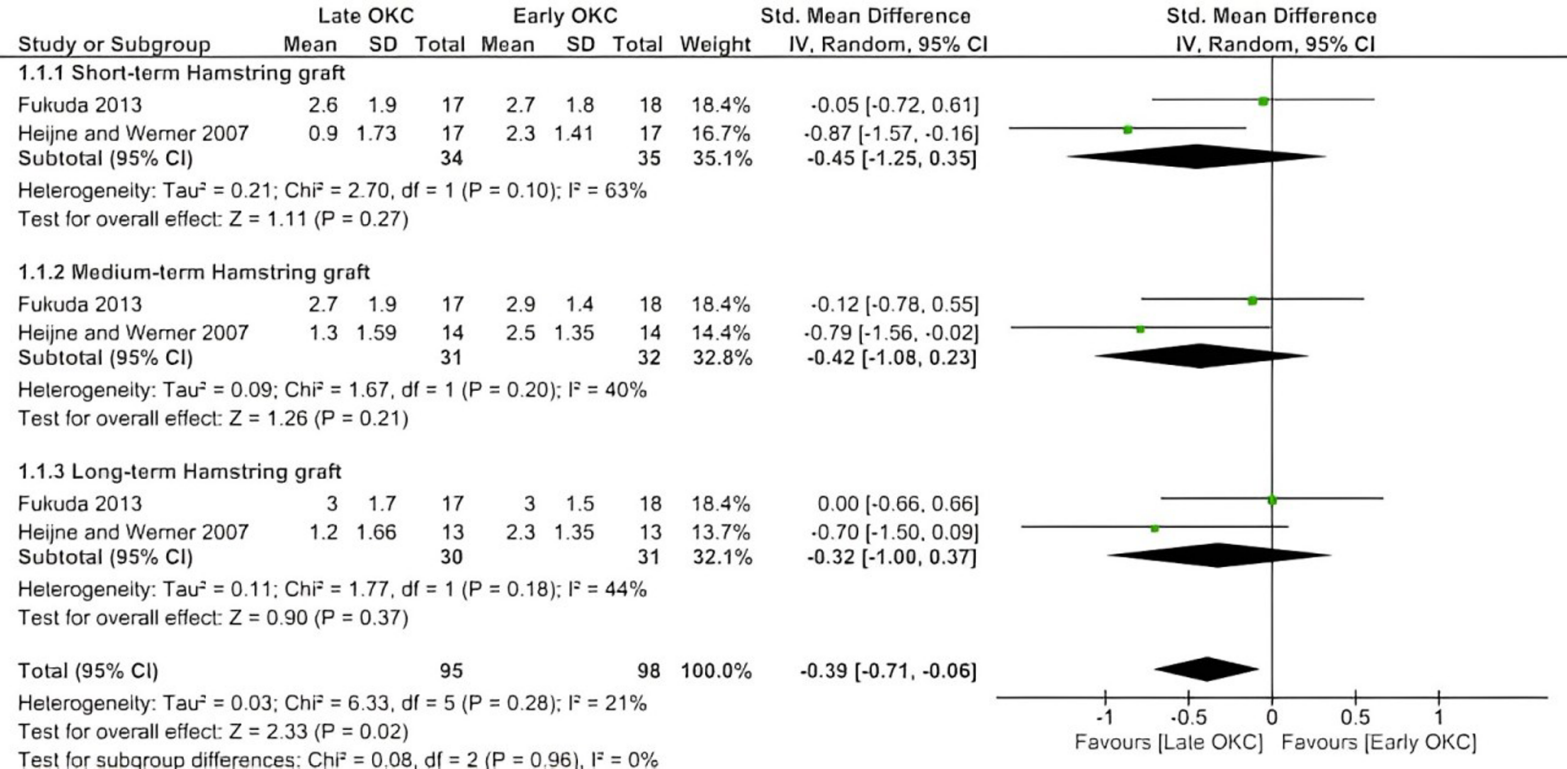
Forest plot showing the effects of early OKC exercise on knee laxity (short, medium, and long term) compared with late OKC exercises. Data are depicted according to measurement conditions: IV, inverse variance.

In the meta-analysis, the effects of early OKC exercises compared with late OKC exercises on laxity were verified by two studies (43, 44) (Figure 2). The findings were verified based on short-term, medium-term, and long-term outcomes in patients with either patellar or hamstring grafts. Late OKC exercises showed a moderate effect size compared to early OKC exercises (SMD = −0.39, favoring late OKC) with a statistically significant difference (*p* = 0.02). The comparison of early vs. late OKC exercises did not show any superiority of either intervention in the short, medium, or long term.

#### 3.6.4 Effectiveness of OKC resistance exercises in patients with ACL-deficient knees

##### 3.6.4.1 Laxity

One study (45) of high risk of bias evaluated the effect of different training loads (high, low, and standard training) on anterior knee stability in ACL-deficient knees using a KT-2000 arthrometer, with a force of 133 N at 30° knee flexion (injured minus uninjured knee laxity corrected for lateral hamstring activity) at 6 and 12 weeks of follow-up (Table 7).

Based on very low certainty evidence, there was a non-significant difference in laxity between the control and LLRT OKC exercise group at 6 weeks (MD = 0.01) and 12 weeks of follow-up (MD = 0.14) (45) (Table 8). There was very low certainty evidence of a significant difference in laxity in favor of the HLRT OKC exercise group compared to the control group at 6 weeks of follow-up (MD = 0.40) and very low certainty evidence of a non-significant difference at 12 weeks of follow-up (MD = 0.19) (45) (Table 11).

**Table 11:** Summary of evidence for the effects of OKC exercises in patients with ACL-deficient knees.

Outcome measure	Comparisons		Relative effect [95% CI]	COKC/OKC (*n* studies)	Quality of evidence (GRADE)	Evidence and significance
	Average estimate in group 1	Average estimate in group 2				
Laxity 6 weeks	Control OKC: Mean ± SD was 0.77 ± 0.43	LLRT OKC: Mean ± SD was 0.76 ± 0.62	MD 0.01 [−0.44, 0.46] Non-statistically significant difference	12/18 (1)	⊕◯◯◯ Very low^a^	Very low certainty evidence of a non-significant difference in laxity between Control and LLRT OKC at 6 weeks of follow-up.
Laxity 6 weeks	Control OKC: Mean ± SD was 0.77 ± 0.43	HLRT OKC: Mean ± SD was 0.37 ± 0.36	MD 0.40 [0.07, 0.73] Non-statistically significant difference	12/10 (1)	⊕◯◯◯ Very low^a^	Very low certainty evidence of a non-significant difference in laxity in favor of HLRT compared to the control group at 6 weeks of follow-up.
Laxity 6 weeks	LLRT OKC: Mean ± SD was 0.76 ± 0.62	HLRT OKC: Mean ± SD was 0.37 ± 0.36	MD 0.39 [−0.05, 0.83] Non-statistically significant difference	18/10 (1)	⊕◯◯◯ Very low^a^	Very low certainty evidence of a non-significant difference in laxity between Control and HLRT OKC at 6 weeks of follow-up.
Laxity 12 weeks	Control OKC: Mean ± SD was 0.71 ± 0.43	LLRT OKC: Mean ± SD was 0.57 ± 0.62	MD 0.14 [−0.31, 0.59] Non-statistically significant difference	12/10 (1)	⊕◯◯◯ Very low^a^	Very low certainty evidence of a non-significant difference in laxity between Control and LLRT OKC at 12 weeks of follow-up.
Laxity 12 weeks	Control OKC: Mean ± SD was 0.71 ± 0.43	HLRT OKC: Mean ± SD was 0.52 ± 0.48	MD 0.19 [−0.18, 0.57] Non-statistically significant difference	12/10 (1)	⊕◯◯◯ Very low^a^	Very low certainty evidence of a non-significant difference in laxity between Control and HLRT OKC at 12 weeks of follow-up.
Laxity 12 weeks	LLRT OKC: Mean ± SD was 0.57 ± 0.62	HLRT OKC: Mean ± SD was 0.52 ± 0.48	MD 0.05 [−0.44, 0.54] Non-statistically significant difference	10/10 (1)	⊕◯◯◯ Very low^a^	Very low certainty evidence of a non-significant difference in laxity between LLRT OKC and HLRT OKC at 12 weeks of follow-up.

GRADE, Grading of Recommendations Assessment, Development and Evaluation; COKC, control open kinetic chain, HLRT, high load resistance training; LLRT, low load resistance training; OKC, open kinetic chain; MD, mean difference; SD, standard deviation; SMD, standardized mean difference.

^a^ Downgraded due to risk of bias.

^b^ Downgraded due to inconsistency.

^c^ Downgraded due to indirectness.

^d^ Downgraded due to imprecision.

Additionally, based on low certainty evidence, there was a non-significant difference in laxity between the control and HLRT OKC exercise group at 6 weeks (MD = 0.39) and 12 weeks of follow-up (MD = 0.05) (45) (Table 11).

## 4 Discussion

### 4.1 Review of results

This systematic review aimed to analyze whether OKC or CKC exercises were more effective in ACL-deficient and reconstructed individuals. Multiple outcome measures were analyzed, including knee laxity, function, self-report function questionnaires, and muscle strength. The secondary aim was to establish the optimal stage of rehabilitation of OKC exercises, with or without resistance, in individuals post-ACLR or with ACL deficiency and determine whether OKC could contribute to reducing anterior laxity of the knee. This study updates the current evidence of previous systematic reviews by including nine studies that evaluated a greater number of outcome measures. While other systematic reviews exist in the literature on this topic, they have not grouped participants into different groups (as in this study), and some even compared dissimilar outcome measures, which increases the risk of bias (14).

### 4.2 OKC vs. CKC exercises in patients after ACLR

Four RCTs (93 participants) compared OKC exercises with CKC exercises in patients after ACL that measured differences in knee laxity, quadriceps strength, function, and pain (37–40). The evidence from these RCTs was inconsistent. The early advantage observed for CKC in Lysholm scores at 2 weeks could suggest potential functional benefits in the initial rehabilitation (short-term) phase (37). However, taking into account the opposite findings, where the authors found statistically significant changes in IKDC form in favor of OKC exercises at a similar time point, raises doubts about the certainty of the evidence of the superiority of the one type of exercise over the other in relation to knee functional assessment in the initial phase (37).

The sustained advantage for CKC exercises at the end of the 6th month indicates a long-term benefit favoring these exercises (40). Similarly, Bynum et al. (62) found significantly higher values regarding Lysholm scores in the CKC group compared to those in the OKC group at 19 months post-surgery. The findings of these studies contradict those of Hooper et al. (51), who found no differences between the OKC and CKC groups in the 4th week. Since the postoperative measurements in the above studies were conducted at later times, it is conceivable that performing the exercises for longer durations could yield more favorable outcomes. Conversely, OKC exercises exhibited an early advantage in specific functional tasks, as indicated by the IKDC Form at 2 weeks (37).

Quadriceps isokinetic strength favors OKC exercises compared to CKC exercises at 12 weeks (38). Mikkelsen et al. (63) found that OKC exercise combined with CKC exercise introduced from 6 weeks post-surgery significantly improved quadriceps strength more than those in CKC exercise at 6 months. Similarly, a rehabilitation program that combined CKC exercise with early OKC exercise significantly improved quadriceps strength at 3 and 6 months on isokinetic testing compared with a rehabilitation program exclusively carried out with CKC exercise (64).

Our meta-analysis showed that OKC exercises could induce higher quadriceps isokinetic strength compared to CKC exercises when performed for 12 weeks. However, there was a lack of information showing the exact postoperative period in which participants were introduced to the protocol (38). Similarly, consistent evidence shows that OKC exercise combined with CKC exercise could significantly improve quadriceps strength compared to only CKC exercise if introduced in the second or sixth postoperative week (21, 63). This can be hypothesized to be due to the enhanced activation and neural drive effects of OKC exercises (65).

The findings of the present study suggest that introducing OKC exercises in ACLR rehabilitation does not significantly increase ACL graft laxity at 14 weeks postoperatively, suggesting that both OKC and CKC contribute similarly to joint stability (39). Bynum et al. (62) found a significant difference in anterior tibial translation between the OKC and CKC groups at 19 months. However, it is difficult to conclude that the differences were due to training, as no knee laxity testing was performed in the period between surgery and the start of rehabilitation. Furthermore, Beynnon and Fleming (66) have also shed doubt on whether the exercises differ in the strain placed on the ACL. As the fixation site becomes stronger, the graft tissue gradually becomes weaker until reaching its weakest point at approximately 12 weeks after surgery, when strength recovery begins (67, 68). Bynum et al. (62) suggested that unrestricted OKC exercises might place too much strain on the ACL graft. Therefore, they recommended that OKC training be performed under controlled conditions and start from week 6 after the ACL reconstruction. However, recently published evidence refutes these results about whether OKC exercises could increase knee laxity and place the graft at a higher risk if they were used in the very early stage of rehabilitation (21, 69). Forelli et al. (21) found no differences at the early initiation of OKC along with CKC exercises at a 90°–0° ROM. The protocol in this study included OKC exercises without the use of external resistance in a period of 2–4 weeks post-surgery and was compared to a rehabilitation program which included only CKC exercises. On the other hand, Wang et al. (69) found that OKC exercises performed without external resistance and in limited ROM (>30°) could be a safer option at the initial stage of rehabilitation. However, the authors used a healthy sample to examine these effects. Despite the appearance of very promising findings, more studies are needed to establish the effect of OKC exercises on anterior knee laxity at the very early stage of rehabilitation.

Function and functional performance, measured by the Hughston knee score and various hop tests at 14 weeks, demonstrated comparable results between the two exercise modalities (39). Similarly, Hooper et al. (70) reported no statistically significant difference between OKC and CKC exercises at 2 and 6 weeks.

Additionally, pain management considerations highlight lower pain associated with CKC exercises at 3 months, suggesting a potential benefit in early rehabilitation (40). The absence of significant differences in pain at 6 months indicates the potential for pain reduction with both types of exercises over the long term (39, 40). Similarly, Morrissey et al. (71) found no significant difference in pain between the groups at 2 and 6 weeks after ACLR surgery using visual analog scales in a self-assessment questionnaire and during maximal isometric contractions of the knee extensors.

### 4.3 OKC vs. CKC exercises in patients with ACL deficiency

The results in individuals with ACL deficiency were drawn from two studies (90 participants) that measured differences in knee laxity and quadriceps strength (19, 41). The quality of evidence for all these outcomes was rated as very low certainty, implying that the results are highly uncertain and should be interpreted cautiously. Our metanalysis suggests that OKC exercise appears to be superior or equally effective to CKC exercise for improving knee laxity as a part of a conservative rehabilitation protocol. Specifically, at 10 and 16 weeks, OKC exercises demonstrated a statistically significant reduction in laxity, as measured by the Knee Signature System device by applying a force of 178 N at 25° flexion and Lachman test 90 N compared to CKC exercises (41).

There was also observed improvement in isokinetic quadriceps muscle strength for OKC exercises compared to CKC exercises at the 4-month follow-up (19). The function of knee extensors after ACL injury is a critical factor in the patient's ability to overcome an injury. Therefore, the use of OKC exercises is important to regain strength and full functionality, taking into account the crucial role of quadriceps as the most important stabiliser of knee joint (72). However, other outcomes, including different outcome measures of knee laxity (Lachman test at 134 N), Hughston knee score, and various hop tests, did not exhibit statistically significant differences between the OKC and CKC groups (41). The Lysholm score (42) and single-legged performance tests also failed to show significant disparities at the 4-month assessment (41). Further research is needed to draw more robust conclusions.

### 4.4 Early vs. late start of OKC exercises in patients after ACLR

The results of early vs. late start of OKC exercises in individuals after ACLR were drawn from two studies (115 participants) that measured differences in knee laxity, quadriceps strength, function and pain (43, 44). The examination of graft laxity, both for patellar and hamstring grafts, revealed consistently very low certainty evidence across short-, medium-, long-, and very long-term follow-ups. Combining data from the two studies in a meta-analysis revealed moderate-quality evidence indicating differences in laxity between early and late OKC exercises when the exercises were introduced earlier than 6 weeks post-ACLR for hamstring grafts. There was no superiority of between-group differences in laxity at short-, medium-, and long-term follow-up. It is crucial to note that the pooled results should not be interpreted as an endorsement for the indiscriminate use of any OKC quadriceps exercises in patients following ACLR, irrespective of graft type. The studies adhered to distinct exercise protocols, revealing disparate responses based on the specific graft used. In one trial in which ACLRs were completed using patellar grafts, the start of OKC exercises occurred 4 weeks post-surgery (44). The progression rate to achieve full extension and the dosage of exercises administered also exhibited variability across the trials. Despite these differences in protocols, no differences in laxity were observed. This is consistent with other studies, which started OKC exercises at 2 weeks (39), 3 weeks (62), or between 6 and 12 weeks and found no increases in laxity for the OKC exercise group (39, 63). Studies employing hamstring grafts for ACLR yielded similar results. Specifically, Heijne and Werner (44) identified no significant difference in laxity (in early OKC exercises) compared to late OKC exercises. Similarly, the study conducted by Fukuda et al. (43) reported no discernible difference in laxity between early OKC and late OKC exercises using hamstring grafts. Combining data from these two studies in a meta-analysis revealed moderate-quality evidence for the hamstring graft with a significant difference between groups in laxity at any time point when OKC exercises were introduced at 4 weeks post-ACLR compared to late OKC exercises. Although the overall effect is statistically significant, the MDs are within error for a device such as the KT-1000. The biggest difference in Heijne and Werner (44) is 1.4 mm (0.9 vs. 2.3 mm), which is below the clinically meaningful threshold of 2 mm and has been associated with increased ACLR graft rupture (73). This is a very small difference in laxity, and although it is statistically significant, it doesn't necessarily mean it is clinically meaningful. Therefore, further research is needed before reaching firm conclusions about the relative safety of the rehabilitation protocols.

Interestingly, our results indicated a significant reduction in pain levels (NPRS) favoring early OKC across short to very long-term follow-ups (43). However, Heijne and Werner (44), using a modified anterior knee score, found that early introduction of OKC exercise had no effect on anterior knee pain in the short, medium, or long term.

Outcomes for quadriceps strength, patient-reported function, and functional performance provided limited evidence demonstrating no between-group differences at any time point with the introduction of OKC exercises in the short, medium, long, and very long term (43). This implies that the timing of OKC exercises may not substantially impact these outcome measures in patients undergoing treatment for ACL injuries.

### 4.5 The effectiveness of OKC resistance exercises in patients with ACL-deficient knees

The findings indicate that at 6 and 12 weeks post-ACLR, early OKC exercise interventions do not appear to significantly impact knee laxity compared to control exercises (45). While there is a suggestion of a potential benefit for HLRT OKC exercises at 6 weeks (moderate effect), the low certainty of the evidence necessitates cautious interpretation. Considering these results within the broader context of ACLR rehabilitation is essential, as is acknowledging the potential variations in patient profiles, surgical techniques, and exercise protocols.

### 4.6 Comparison with systematic reviews and meta-analyses

Previous systematic reviews and meta-analyses of exercises in patients after ACLR have consistently concluded that there is no significant superiority of one type of exercise (OKC or CKC) over the other in patients after ACLR (14, 74, 75). Additionally, the systematic reviews of Glass et al. (76) and Wright et al. (77) have recommended introducing OKC exercises from the 6th week post-ACL rehabilitation. This is further supported by Andersson et al. (78). In addition, OKC exercises can be initiated as early as 4 weeks post-operation, albeit within a restricted ROM between 90° and 45° (76, 77). The systematic review of systematic reviews by Lobb et al. (75) concluded that there is limited evidence to support the use of a combination of OKC and CKC quadriceps exercises. This combination did not appear to improve strength and return to play compared to CKC exercises alone. Furthermore, there are no differences in pain, function, and laxity when comparing OKC to CKC exercises during ACL reconstruction rehabilitation.

Perriman, Leahy and Semciw (14) compared OKC and CKC exercises in ACLR patients and discovered that both types yielded similar results in terms of strength, function, and anterior knee laxity, whether initiated early or late. In their review, they used 10 RCTs, and the results (low to moderate evidence) showed no significant difference between the two types of exercise in terms of strength, function, and anterior knee laxity in both the early and late start of exercises. Limited data suggested that early OKC quadriceps exercises might have less affected patellar tendon grafts. Jewiss et al. (74) found no significant difference in clinical outcomes between OKC and CKC exercises in ACLR patients based on data from seven RCTs. This systematic review used seven RCTs, and the results showed that both types of exercise could be effective in the rehabilitation of patients after ACLR since, as previously mentioned, no evidence was found that one form of exercise is superior to the other.

Glass et al. (76) reported that CKC and OKC exercises had similar effects on knee laxity, pain, and function in patients with ACL deficiency or reconstructions. They recommended a conservative approach, continuing with CKC exercises, given limited research on potential risks associated with OKC exercises.

Trees et al. (79) compared CKC and OKC exercises using seven RCTs. They observed no significant differences in knee function, pain, or laxity in CKC vs. OKC trials. In CKC vs. combined CKC and OKC exercises, a greater return to pre-injury sports levels was noted at 31 months, with no distinctions in secondary measures of strength and knee laxity at 6 months.

Unlike previous systematic reviews, this one stands out by encompassing a broader scope (14, 74, 78). It included nine RCTs and extended its examination beyond the realms of OKC and CKC exercises exclusively in patients after ACLR. Instead, it delved into comparisons between OKC and CKC exercises in patients with and without ACLR, scrutinized the early vs. late introduction of OKC exercises post-ACLR, and assessed the efficacy of resistance-based OKC exercises in patients with ACL deficiency. This extensive approach distinguishes the present systematic review from its predecessors.

### 4.7 Limitations

The gender distribution within the included studies was imbalanced, primarily featuring more male participants, which can limit the generalizability of findings and overlook potential gender-specific differences. Not all studies examined all the outcome measures but also mean and standard deviations for some outcomes of interest were not reported in the articles, potentially leading to incomplete data and a limited assessment of the overall impact of OKC and CKC exercises. Specific limitations were identified within individual studies, such as the lack of preoperative knee laxity measurements and potential biases related to surgical procedures. Variability in exercise protocols, including sets, repetitions, and resistance levels, among the included studies may affect the comparability of outcomes and the generalizability of results. The evidence quality in some cases was rated as very low or low certainty, emphasizing the need for more high-quality research to draw definitive conclusions. Other unaccounted factors, such as graft type or individual patient characteristics, could influence the effectiveness of OKC and CKC exercises. Furthermore, as stated in the methodology, published data in predatory journals were not retrieved or considered in the meta-analysis. Hence, we cannot comment on whether the inclusion of additional data from these journals may change the findings and interpretations.

### 4.8 Suggestions for future research

Several suggestions for future research emerge. Firstly, future research should investigate the long-term effects of OKC and CKC exercises in patients with ACL deficiency and reconstruction, especially on knee laxity, function, functional performance, and muscle strength. To provide more robust evidence, future studies should recruit larger sample sizes and use additional follow-up periods. Secondly, assessing the optimal timing and dosage of OKC exercises, especially in combination with CKC exercises, may enhance rehabilitation outcomes and significantly limit the risk of complications. Additionally, examining potential gender-specific differences in response to rehabilitation exercise programmes may contribute to more individualized treatment approaches. Thirdly, researching the impact of variations in exercise protocols, including sets, repetitions, and resistance, on outcome measures can provide valuable insights into refining rehabilitation strategies. Lastly, investigating the efficacy of OKC resistance exercises, particularly in ACL-deficient patients, and their effects on pain management and functional outcomes warrants further exploration to optimize rehabilitation protocols.

## 5 Conclusion

Very low certainty of evidence suggests that OKC exercises appear to be superior to CKC for improving quadriceps strength at 3–4 months after injury, either as a part of conservative or post-surgery rehabilitation. Very low certainty of evidence showed inconsistent results between OKC and CKC exercises in relation to outcome measures dealt with knee function in ACLR. However, CKC exercises showed a significant superiority in knee function at 6 weeks post-ACLR. On the other hand, OKC exercise seems to be either superior or equally effective to CKC exercise for improving knee laxity, thus presenting their importance in being included in a rehabilitation protocol from the initial phase, but these findings should be interpreted with caution. Concerns about graft strain with OKC exercise necessitate cautious implementation from 4 weeks post-ACLR. Finally, very low certainty of evidence suggests a significant decrease in pain in ACLR in favor of early OKC exercises compared to late CKC exercises.

## Data Availability

The original contributions presented in the study are included in the article, further inquiries can be directed to the corresponding author.
